# T4 stage and preoperative anemia as prognostic factors for the patients with colon cancer treated with adjuvant FOLFOX chemotherapy

**DOI:** 10.1186/s12957-015-0488-7

**Published:** 2015-02-19

**Authors:** Min Sung An, Jong Han Yoo, Kwang Hee Kim, Ki Beom Bae, Chang Soo Choi, Jin Won Hwang, Ji Hyun Kim, Bo Mi Kim, Mi Seon Kang, Min Kyung Oh, Kwan Hee Hong

**Affiliations:** Department of Surgery, Busan Paik Hospital, Inje University College of Medicine, 75 Bokji-ro, Busanjin-gu, Busan 614-735 Korea; Internal medicine, Busan Paik Hospital, Inje University College of Medicine, 75 Bokji-ro, Busanjin-gu, 614-735 Korea; Pathology, Busan Paik Hospital, Inje University College of Medicine, 75 Bokji-ro, Busanjin-gu, 614-735 Korea; Clinical Trial Center in Pharmacology, Busan Paik Hospital, Inje University College of Medicine, 75 Bokji-ro, Busanjin-gu, 614-735 Korea

**Keywords:** Colon cancer, FOLFOX, T4 stage, Anemia

## Abstract

**Background:**

FOLFOX-based adjuvant chemotherapy is a benefit for high-risk stage II and stage III colon cancer after curative resection. But, the prognostic factor or predictive marker for the efficacy of FOLFOX remains unclear. This study was aimed to identify the prognostic value and cumulative impact of adjuvant FOLFOX on the stage II and III colon cancer patients.

**Methods:**

A total of 196 stage II and III colon cancer patients were retrospectively enrolled in prospectively collected data. They underwent curative resection followed by FOLFOX4 adjuvant chemotherapy. The oncological outcomes included the 5-year disease-free survival (DFS) rate and 5-year overall survival (OS) rate. Cox-regression analysis was performed to identify the prognostic value, and its cumulative impact was analyzed.

**Results:**

The 5-year DFS rate of the patients was 71.94% and the 5-year OS rate was 81.5%. The prognostic values for the 5-year DFS rate and 5-year OS rate were T4 stage and preoperative anemia in a multivariate analysis. Each patient group who had no prognostic value, single, or both factors revealed 95.35%, 69.06%, and 28.57% in the 5-year DFS rate, respectively (*p* < 0.0001). The 5-year OS rate also showed the significant differences in each group who had no prognostic value, single, or both factors revealed 100%, 79.3%, and 45.92%, respectively (*p* < 0.0001).

**Conclusion:**

Our results showed similar efficacy to MOSAIC study in stage II and stage III colon cancer patients treated with adjuvant FOLFOX chemotherapy after curative resection. Patients who had T4 stage and/or preoperative anemia showed worse prognosis than patients without any prognostic value. These findings suggest that FOLFOX could not be effective in the patients with T4 stage colon cancer accompanied by preoperative anemia.

## Background

Colorectal cancer is the second most common cancer in the USA and the third most common cancer in Korea [1]. The most important treatment for colon cancer is surgical resection. However, 40% to 50% of these postsurgical patients eventually experience recurrence or die from metastatic lesions [2,3]. In the 1990s, O’Connell *et al.* [4] reported low recurrence and mortality rates in patients with stage III colon cancer who had received 5-fluorouracil (5-FU) and low-dose leucovorin (LV) injections as chemotherapy after surgical resection. The Intergroup Trial (INT-0035) reported that administration of 5-FU and levamisole injections as adjuvant chemotherapy after surgery in patients with stage III colon cancer decreases the mortality rate by 33% [5]. Subsequently, many reports suggested that a 5-FU and LV combination became the standard adjuvant treatment for stage III colon cancer [6,7].

The MOSAIC (Multicenter International Study of Oxaliplatin/5-FU/LV in the Adjuvant Treatment of Colon Cancer) trial reported that the FOLFOX chemotherapeutic regimen, for which oxaliplatin (a third-generation platinum derivative alkylating agent) was added to 5-FU and LV, showed a superior disease-free survival (DFS) rate than the 5-FU and LV (LV5FU2) regimen [8,9]. Additional follow-up observations of the MOSAIC trial continuously demonstrated that FOLFOX chemotherapy is advantageous in terms of both the DFS and overall survival rates [9]. Thus, the 2013 National Comprehensive Cancer Network (NCCN) guidelines recommend FOLFOX4 or XELOX chemotherapy for patients with high-risk stage II and stage III colon cancer after surgery [10].

However, the 5-year survival rate from each stage, American Joint Committee on Cancer (AJCC) sixth edition staging, showed paradoxically the lower survival rate in stage IIb (72.2%) than in stage IIIa (83.4%) [3]. There should be several poor prognostic factors affecting the survival rate even after the adjuvant chemotherapy. FOLFOX-based adjuvant chemotherapy is a benefit for high-risk stage II and stage III colon cancers after curative resection. But, the prognostic factor or predictive marker for the efficacy of FOLFOX remains unclear. This study was aimed to identify the prognostic value and cumulative impact of adjuvant FOLFOX on the stage II and III colon cancer patients.

## Methods

### Subjects

This retrospective study included 196 patients with colon cancer who were administered FOLFOX4 chemotherapy after radical surgery in the Department of Surgery, Busan Paik Hospital, Inje University College of Medicine between April 2006 and December 2010. The stage of colon cancer were classified in accordance with the sixth edition of the AJCC TNM staging system, and the high-risk stage II and III patients who had been treated with adjuvant FOLFOX chemotherapy were enrolled.

Colon cancer was defined as cancer in which the lower tumor margin was located in the upper part of the peritoneum, and the stage II high-risk group must have at least one of the following factors, including T4a/4b, tumor perforation, bowel obstruction, poorly differentiated tumor, or venous, perineural, or lymphatic invasion. We investigated not only the postsurgical pathological characteristics but also the ASA score and preoperative laboratory findings, which reflected the general state of patients before undergoing treatment, as well as the adverse reactions that developed during chemotherapy. Adverse reactions were examined by dividing them into three categories: 1) neutropenia (cases with grade 3 or 4), 2) gastrointestinal symptoms (diarrhea, patients prescribed loperamide, nausea or anorexia), and 3) peripheral neuropathy (patients prescribed gabapentin). Written informed consent was obtained from the patient for the publication of this report and any accompanying images.

### Chemotherapy method and follow-up observations

LV 200 mg/m^2^/day was administered intravenously for 2 h. Then, a bolus IV of 5-FU 400 mg/m^2^ was administered, which was followed by intravenous administration of 5-FU 600 mg/m^2^ continuously for the remaining 22 h. This regimen was continued for 2 days. Oxaliplatin 85 mg/m^2^ was infused for 2 h only on day 1. A prophylactic antiemetic and sufficient fluid were infused on days 1 and 2 of chemotherapy. This regimen was administered every 2 weeks. The adjuvant chemotherapeutic regimen was carried out for a total of 12 cycles.

Patients were followed up every 3 months for the first 2 years after surgery and every 6 months thereafter for 3 years, for a total of 5 years of follow-up. History, physical examination, and serum carcinoembryonic antigen levels were determined at each follow-up visit. Chest X-ray and abdominopelvic computed tomography scans were performed to assess the efficacy of chemotherapy every four cycles and every 6 months after completion of chemotherapy. A colonoscopy was performed annually. Recurrence was identified by imaging studies and colonoscopy and was confirmed by colonoscopic or percutaneous biopsy. Radiologically identified tumor growth within the previous surgical field was considered to indicate recurrence when histological confirmation was not possible.

### Statistical analysis

This study was the observational setting. The oncologic outcome was analyzed with 5-year DFS and 5-year overall survival (OS) rate. Each survival rate was analyzed with the Kaplan-Meier method. Cox proportional hazards model was used for the univariate and multivariate analyses of factors affecting the prognosis. The Kaplan-Meier method and log-rank test were conducted to compare the DFS and OS rates among risk groups. A *p* value <0.05 was considered to indicate significance. SAS ver. 9.3 (SAS Institute, Cary, NC, USA) was used for the statistical analysis.

## Results

### Clinicopathological data

The mean follow-up period was 61 ± 31 months, and among the 219 patients, 23 patients had been lost during their follow-up. A total of 196 patients were included [age range 26 to 76 years, median age 57 years, 112 males (57.1%) and 84 females (42.9%)]. In total, 147 patients (75%) were <65 years, and 49 patients (25%) were ≥65 years. A total of 174 patients (88.8%) belonged to the category of moderately differentiated tumors and 174 patients (88.8%) belonged to the T3 stage, whereas 86 patients (43.9%) were in the N1 stage. Of the 196 patients, 158 patients completed a total chemotherapy of 12 cycles. Thirty-eight patients did not complete all chemotherapy cycles due to neutropenia, gastrointestinal symptoms (diarrhea, nausea, or anorexia), peripheral neuropathy, or cancer recurrence. Of the 196 patients, 154 (78.6%) had neutropenia, 36 (18.4%) had gastrointestinal symptoms, and 40 (20.4%) had peripheral neuropathy (Table 1).

**Table 1:** **Clinicopathologic characteristics of the patients**

**Characteristic**	**Number**	**%**
Total patients	196	
Median age (range)	57 (26 to 76)	
<65	147	75.0
≥65	49	25.0
Sex		
Male	112	57.1
Female	84	42.9
Tumor location		
Right	52	26.5
Transverse	8	4.1
Descending	17	8.7
Sigmoid and rectosigmoid	119	60.7
Histologic appearance		
Well differentiated	6	3.1
Moderately differentiated	174	88.8
Poorly differentiated	10	5.1
Signet ring cell	1	0.5
Mucinous cell	5	2.5
Lymphovascular invasion (+)	106	54.1
Neural invasion (+)	89	45.4
T stage		
T1	1	0.5
T2	5	2.5
T3	174	88.8
T4	16	8.2
N stage		
N0	38	19.4
N1	86	43.9
N2	72	36.7
WBC		
<4,000 × 10^6^/L	3	1.5
4,000 ~ 10,000 × 10^6^/L	169	86.2
>10,000 × 10^6^/L	24	12.3
Hemoglobin (Hgb)		
Female <12 g/dL (anemia)	50	25.5
Female ≥12 g/dL (normal)	34	17.3
Male <14 g/dL (anemia)	66	33.7
Male ≥14 g/dL (normal)	46	23.5
Platelet (PLT)		
<140 × 10^9^/L (thrombocytopenia)	4	2.0
140 ~ 440 × 10^9^/L	185	94.4
>440 × 10^9^/L	7	3.6
Albumin		
<3.8 g/dL	40	20.4
3.8 ~ 5.3 g/dL	156	79.6
Lactate dehydrogenase (LDH)		
129 ~ 240 U/L	41	20.9
>240 U/L	155	79.1
Total bilirubin		
0.2 ~ 1.0 mg/dL	189	96.4
>1.0 mg/dL	7	3.6
Aspartate aminotransferase (AST)		
10 ~ 33 U/L	174	88.8
>33 U/L	22	11.2
Alanine aminotransferase (ALT)		
4 ~ 50 U/L	183	93.4
>50 U/L	13	6.6
Protein		
<6.7 g/dL	29	14.8
6.7 ~ 8.3 g/dL	165	84.2
>8.3 g/dL	2	1.0
Prognostic model (T4, Hgb)		
Low-risk group	43	21.9
Intermediate-risk group	139	70.9
High-risk group	14	7.2
Preoperative CEA		
≤5 ng/mL	114	58.2
>5 ng/mL	82	41.8
Postoperative CEA		
≤5 ng/mL	160	81.6
>5 ng/mL	36	18.4
Side effect		
Neutropenia	154	78.6
Gastrointestinal symptoms	36	18.4
Peripheral neuropathy	40	20.4

CEA, carcinoembryonic antigen; WBC, white blood cell.

### Analysis of survival rates (DFS, OS) and prognostic factors

**Figure 1:**
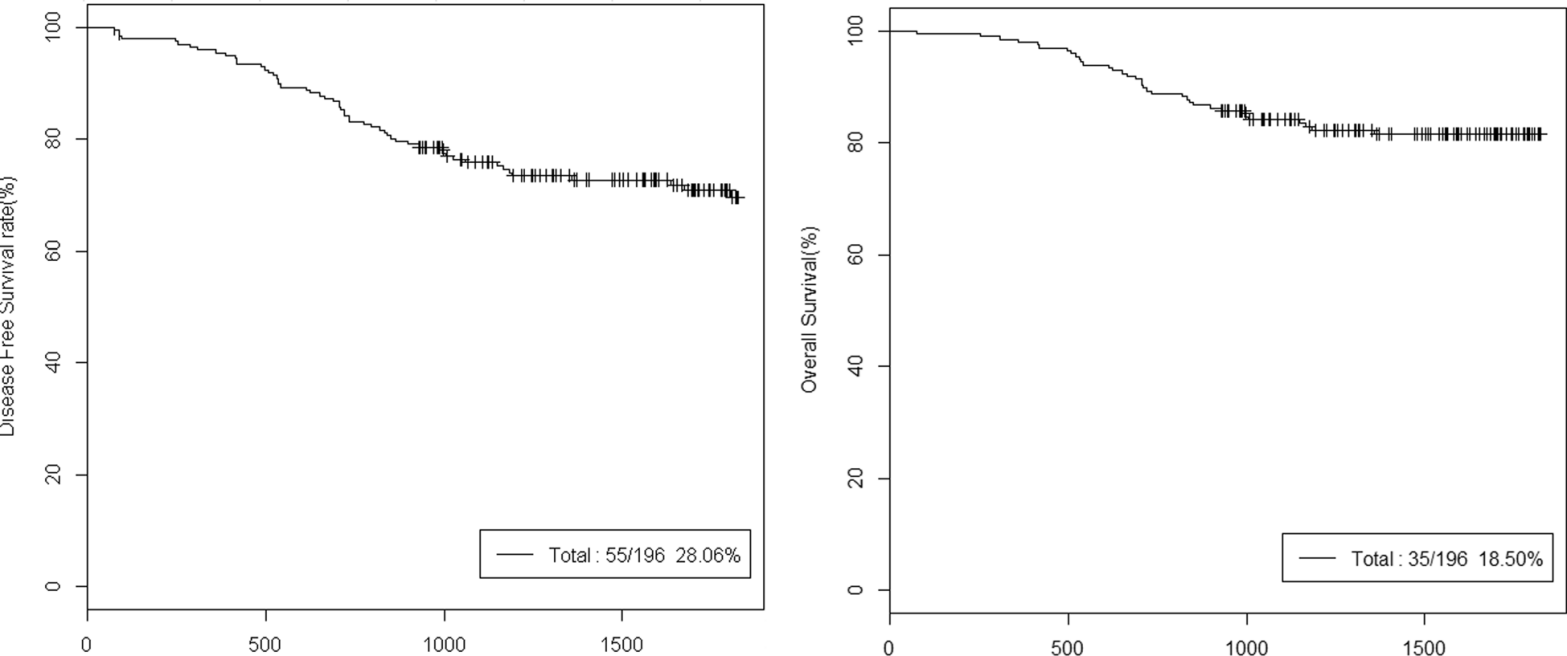
**5-year DFS and OS of total patients.** DFS, disease free survival; OS, overall survival.

**Figure 2:**
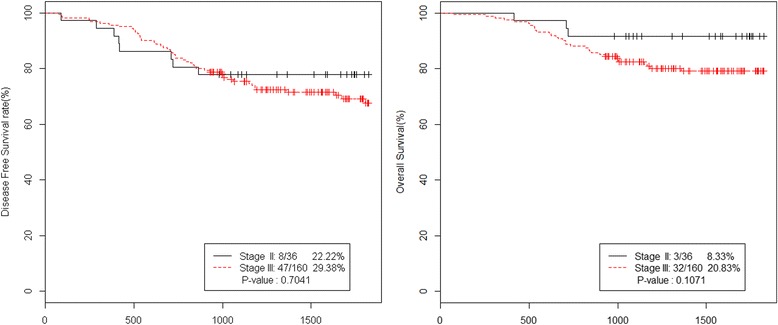
**5-year DFS and OS of stage II vs. stage III.** DFS, disease free survival; OS, overall survival.

The 5-year DFS rate of the all patients was 71.94% and the 5-year OS rate was 81.5% (Figure 1). The 5-year DFS rates of the high-risk stages II and III were 77.78% and 70.62%, respectively. And the 5-year OS rates of the high-risk stages II and III were 91.67% and 79.17%, respectively (Figure 2).

In the univariate analysis, prognostic factors for DFS were T4 stage (*p* = 0.0018), preoperative anemia (*p* = 0.0019), and preoperative thrombocytopenia (*p* = 0.0444), and prognostic factors for OS were T4 stage (*p* = 0.005), preoperative anemia (*p* = 0.0143), preoperative thrombocytopenia (*p* = 0.0485), postoperative carcinoembryonic antigen (CEA) (*p* = 0.0019), and low albumin level (*p* = 0.0298) (Table 2). Statistically significant factors in the univariate analysis were included in a multivariate analysis.

**Table 2:** **Univariate analysis of prognostic factors for DFS and OS**

**Factor**	**Classification**	***p*** **value (DFS)**	***p*** **value (OS)**
Sex	Female		
	Male	0.9818	0.8802
Age	<65		
	≥65	0.5093	0.8196
Cancer location	Right		
	Transverse	0.2048	0.0534
	Descending	0.2056	0.5503
	Sigmoid and rectosigmoid	0.7813	0.9634
Lymphovascular invasion	Negative		
	Positive	0.4643	0.2176
Neural invasion	Negative		
	Positive	0.3234	0.6679
Histology (differentiation )	Well		
	Moderately	0.5575	0.9728
	Poorly	0.9001	0.894
	Signet ring cell	0.1424	0.1516
	Mucinous cell	0.8841	0.8773
WBC	4,000 ~ 10,000 × 10^6^/L		
	<4,000 × 10^6^/L	0.8888	0.9328
Hgb	Normal		
	Anemia	0.0019	0.0143
PLT	140 ~ 440 × 10^9^/L		
	Thrombocytopenia	0.0444	0.0485
	>440 × 10^9^/L	0.6451	0.7521
Albumin	3.8 ~ 5.3 g/dL		
	<3.8 g/dL	0.3055	0.0298
LDH	129 ~ 240 U/L		
	>240 U/L	0.1801	0.2756
Total bilirubin	0.2 ~ 1.0 mg/dL		
	>1.0 mg/dL	0.2247	0.8442
AST	10 ~ 33 U/L		
	>33 U/L	0.1258	0.4506
ALT	4 ~ 50 U/L		
	>50 U/L	0.4121	0.7752
Protein	6.7 ~ 8.3 g/dL		
	<6.7 g/dL	0.3451	0.3746
T stage	1		
	2		
	3		
	4	0.0018	0.005
N stage	0		
	1	0.2954	0.4345
	2	0.6658	0.1171
Preoperative CEA	≤5 ng/mL		
	>5 ng/mL	0.4768	0.2144
Postoperative CEA	≤5 ng/mL		
	>5 ng/mL	0.0585	0.0019
ASA score	1		
	2	0.31	0.7768
	3	0.7646	0
Neutropenia	Absent		
	Present	0.7597	0.1927
GI Symptom (diarrhea, nausea, anorexia)	Absent		
	Present	0.6714	0.508

ALT, alanine aminotransferase; ASA, American Society of Anesthesiologists; AST, aspartate aminotransferase; CEA, carcinoembryonic antigen; GI symptoms, gastrointestinal symptoms (diarrhea, constipation, anorexia); Hgb, hemoglobin; LDH, lactate dehydrogenase; PLT, platelet; WBC, white blood cell.

In the multivariate analysis, prognostic factors for DFS were T4 stage (*p* = 0.0032) and preoperative anemia (*p* = 0.0043) (Table 3). And the prognostic factors for OS were T4 stage (*p* = 0.0124), postoperative CEA (*p* = 0.0032), and preoperative anemia (*p* = 0.0313) (Table 4).

**Table 3:** **Multivariate analysis of prognostic factors for DFS**

**Factor**	**Classification**	**Hazard ratio**	**95%** **confidence interval**		***p*** **value**
T stage	1				
	2	0	0		
	3	0	0		
	4	2.747	1.402	5.383	0.0032
Hgb	Normal				
	Anemia	5.505	1.708	17.745	0.0043
PLT	140 ~ 440 × 10^9^/L				
	Thrombocytopenia	3.213	0.976	10.581	0.0549
	>440 × 10^9^/L	1.14	0.273	4.758	0.8574
Postoperative CEA	≤5				
	>5	1.672	0.91	3.073	0.0975
Prognostic model (T4-Hgb)	Low risk				
	Intermediate risk	7.401	1.786	30.67	0.0058
	High risk	19.296	4.197	88.723	0.0001

CEA, carcinoembryonic antigen; Hgb, hemoglobin; PLT, platelet.

**Table 4:** **Multivariate analysis of prognostic factors for OS**

**Factor**	**Classification**	**Hazard ratio**	**95%** **confidence interval**		***p*** **value**
T stage	1				
	2	0	0		
	3	0	0		
	4	2.904	1.259	6.697	0.0124
Hgb	Normal				
	Anemia	9.01	1.219	66.62	0.0313
PLT	140 ~ 440 × 10^9^/L				
	Thrombocytopenia	2.288	1.04	8.882	0.0654
	>440 × 10^9^/L	1.244	0.374	4.136	0.7221
Postoperative CEA	≤5				
	>5	2.961	1.439	6.095	0.0032

CEA, carcinoembryonic antigen; Hgb, hemoglobin; PLT, platelet.

**Figure 3:**
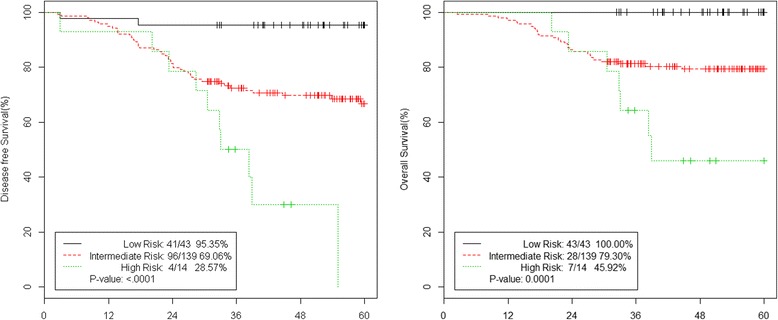
**5-year DFS and OS of low vs. intermediate vs. high**-**risk group.** DFS, disease free survival; OS, overall survival.

Prognostic models were prepared using the two common prognostic factors, T4 stage and preoperative anemia. These prognostic models were classified into the following three groups: 1) low-risk group without either of these prognostic factors, 2) intermediate-risk group with only one prognostic factor, and 3) high-risk group with both prognostic factors. Each patient group who had no prognostic value (low-risk group), single (intermediate-risk group), or both factors (high-risk group) revealed 95.35%, 69.06%, and 28.57% in the 5-year DFS rate, respectively (*p* < 0.0001). The 5-year OS rate also showed the significant differences in each group who had no prognostic value (low-risk group), single (intermediate-risk group), or both factors (high-risk group) revealed 100%, 79.3%, and 45.92%, respectively (*p* < 0.0001) (Figure 3). The multivariate analysis of each risk group for DFS showed that the hazard ratio (HR) of the intermediate-risk group was 7.401 (95% confidence interval (CI) 1.786 to 30.67, *p* = 0.0058) and that of the high-risk group was 19.296 (95% CI 4.197 to 88.723, *p* = 0.0001) (Table 3).

## Discussion

We assessed the 5-year DFS rate, 5-year OS rate, and prognostic factors that affected treatment of patients with colon cancer in the high-risk stage II group and patients in stage III. These patients underwent adjuvant FOLFOX4 chemotherapy after surgery. Numerous studies have reported that adjuvant chemotherapy after radical surgery improves the survival rate of patients with stage III colon cancer [6-9].

In the MOSAIC trial, the 5-year DFS rate for patients with stage III colon cancer, who underwent adjuvant FOLFOX chemotherapy, was 73.3%. This figure was superior to the 67.4% 5-year DFS rate of LV5FU5 chemotherapeutic regimen (HR 0.80, 95% CI 0.68 to 0.93, *p* = 0.003) [8,11]. However, no significant differences were observed between the two groups in the 5-year DFS rate or overall survival rate of patients with stage II colon cancer [8,12]. The National Surgical Adjuvant Breast and Bowel Project (NSABP) C-07 trial compared the oncologic outcomes of FLOX (oxaliplatin + leucovorin + fluorouracil) and FULV (leucovorin + fluorouracil), adjuvant chemotherapeutic regimens, in patients with stage III colon cancer. The 5-year DFS rate of the group treated with FLOX chemotherapy was 69.4%, whereas that of the group treated with FULV chemotherapy was 64.2% (HR 0.82, 95% CI 0.72 to 0.93, *p* < 0.001) [11,13]. We investigated 196 patients, belonging to the high-risk stage II or stage III groups, who underwent adjuvant FOLFOX4 chemotherapy after radical surgery, and found a 5-year DFS rate of 71.94% and 5-year OS rate of 81.5%. The 5-year DFS and 5-year OS rate of the 160 patients in stage III showed 70.62% and 79.17%, separately. This outcome was similar to that of the MOSAIC or NSABP C-07 trials, representative investigations in which oxaliplatin was added to the FULV chemotherapeutic regimen.

There are numerous variables for the candidates for the prognostic factors of FOLFOX chemotherapy. McMillan *et al.* [14] reported that not only objective cancer staging but also nutritional state and factors reflecting systemic inflammation (weight loss, CRP increase, or decreased albumin) affect the prognosis after cancer treatment. Lee *et al*. [15] analyzed prognostic factors in 1,455 patients with progressive gastric cancer, who were treated with taxotere, taxol, FOLFOX, FOLFIRI, or FOLFOXIRI chemotherapeutic regimens. They reported that decreased albumin, increased alkaline phosphatase, bone metastasis, or ascites adversely affected the survival rate. But we chose the three categories affecting adjuvant FOLFOX4 chemotherapy according to 1) patients’ clinical characteristics, 2) preoperative laboratory findings regarding the general condition of patients before undergoing treatment, and 3) postsurgical pathologic features. In this study, the multivariate analysis showed that T4 stage and preoperative anemia were the significant prognostic factors for both DFS and OS. Snaebjornsson *et al*. [16] reported that pT4 stage, among many variables analyzed in patients with stages II and III colon cancer, is the most important indicator of a poor prognosis. They also reported that it had significance equal to that of lymph node status. In a comparison of stages pT4 and pT3 among 352 patients with stage II colon cancer, the 5-year survival rates were 50% and 82%, respectively (HR 2.92, 95% CI 1.67 to 5.10, *p* < 0.001). Gunderson *et al*. [17] reported that pT4N0M0 stage II tumors have a poorer prognosis than pT1-2 N1-2 M0 stage III tumors. Thus, pT4 stage is a rather important independent prognostic factor in the treatment of colon cancer. Pretreatment anemia is associated with poor prognosis in variable cancers such as lung cancer, cervical cancer, head and neck cancer, and esophageal cancer [18-21]. Qiu *et al*. reported that pretreatment anemia (HR 0.084, 95% CI 0.037 to 0.191, *p* < 0.001) and thrombocytosis (HR 3.475, 95% CI 1.564 to 7.721, *p* = 0.002) in colorectal cancer patients might be useful prognostic markers [22]. Anemia in colorectal cancer patients is thought to be due to inflammatory cytokines and occult bleeding, and anemia can cause intratumoral hypoxia [23]. Intratumoral hypoxia might be an important factor in the activation of hypoxia-inducible factor-1, which can contribute in the acceleration of tumor metastasis [24]. We also established a prognostic model using T4 stage and preoperative anemia. The high-risk group (HR 19.296, 95% CI 4.197 to 88.723, *p* = 0.0001) with both factors, T4 stage, and preoperative anemia showed a significantly worse prognosis than the other two groups.

## Conclusion

In conclusion, our results showed similar efficacy to the MOSAIC study in stage II and stage III colon cancer patients treated with adjuvant FOLFOX chemotherapy after curative resection. Patients who had T4 stage and/or preoperative anemia showed worse prognosis than patients without any prognostic value. These findings suggest that FOLFOX could not be effective in the patients with T4 stage colon cancer accompanied by preoperative anemia. Therefore, much caution and aggressive additional adjuvant treatment should be used when treating T4 stage colon cancer patients accompanied by preoperative anemia with FOLFOX-based adjuvant chemotherapy.
